# Physiotherapy Rehabilitation for Above-Knee Amputation Secondary to Infected External Fixation: A Case Report

**DOI:** 10.7759/cureus.51689

**Published:** 2024-01-05

**Authors:** Anushka M Biyani, Neha Arya, Maithili Deshpande, Nandini C Baheti

**Affiliations:** 1 Musculoskeletal Physiotherapy, Ravi Nair Physiotherapy College, Datta Meghe Institute of Higher Education and Research, Wardha, IND

**Keywords:** rehabilitation, talus fracture, fibula, displaced distal tibia, knee amputation

## Abstract

This case study examines the total physiotherapy care of a 50-year-old male patient, who had a right-sided displaced distal tibia and fibula fracture, a talus fracture due to a road traffic accident, and an above-knee amputation due to a serious infection. Enhancing muscle strength, reducing pain from phantom limbs, avoiding problems, maintaining range of motion, increasing endurance, and promoting functional independence in the postoperative period were the main goals of the patient's rehabilitation. The recovery plan included an intensive four-week program of physiotherapy care. The regimen included a variety of interventions, such as pain management, edema control, wound healing techniques, range of motion (ROM) exercises, muscle strengthening activities, mobility and transfer exercises, cardiovascular endurance training, psychosocial support, education on prosthetic use, and independence in daily living activities. ROM measures, manual muscle testing, and functional independence measure scores were used to evaluate the patient's improvement. The patient's physical health and level of functional independence both exhibited significant improvements, according to the statistics. Following treatment, the patient's ROM, muscle strength, and overall functional independence all improved. The study highlights the positive impacts of physical therapy interventions on the patient's quality of life, mobility, and self-sufficiency following the amputation and subsequent recovery. These findings support the patient's transition to a more self-sufficient and active lifestyle by providing valuable insights into the efficient use of physiotherapy and the comprehensive post-amputation treatment plan.

## Introduction

Amputation is the surgical removal of all or a portion of a physical part, such as an arm or limb. Disarticulation, in which a component is extracted through a joint, is often different from it [1,2]. The WHO classification system (ICD) code numbers were utilized to extract the following four etiology groups: vascular insufficiency, diabetes mellitus, malignant neoplasia, and trauma. The goal was to examine the connection between etiology, the reason for the amputation, and the level of amputation (foot, below-knee, through-knee, above-knee, and hip). Throughout the study, fewer people with and without diabetes mellitus underwent amputations due to vascular insufficiency. The number of amputations due to road traffic accidents (RTAs) and tumors appeared to be constant [3]. One of the most frequent reasons for hospital admissions is fractures to the proximal tibia and fibula. According to a study, fractures resulting from RTAs account for 78% of all fractures, with motorcyclists accounting for 42% of these cases [4]. The number of amputations for vascular insufficiency, both with and without diabetes mellitus, at proximal levels above the knee and in the trauma group was much lower. There was no discernible shift in the amputation of tumors [5]. The distribution of the etiological components level showed a distinctive pattern for each cause and for each amputation level [6]. Amputation rates ranged from 14 to 32 per year, with an average of 21.6 per year. The year 2003 saw the most amputations. Patients ranged in age from 3 to 73 years, with an average age of 39.26 ± 12.6 years. Of the total patients,172 (79.62%) were male and 44 (20.37%) were female [7]. Around 27% of lower limb amputations include an above-knee amputation. Although amputations can occur in any age group, they are more common in those aged 65 and older. Above-knee amputations entail chopping through the femoral bone and thigh tissue to remove the limb from the body. Numerous conditions may need this surgery, including trauma, infection, tumors, and vascular impairment [8]. Amputations of the lower limb account for 94.8% of all amputations, whereas amputations of the upper limb account for just 5.2%.

Amputations below the knee are the most common type of lower limb amputation, followed by amputations above the knee. Around 4% of people worldwide live in developing nations, whereas 7% live in developed nations. In India, 2.1-3.0% of people had a disability in 2001. The reasons for amputations are reported differently in different nations. The degree of industrialization, the quality of the medical care system, and the transportation infrastructure all impact the major causes of amputation in various nations. Mortality rates in diabetic lower extremity amputation patients can be as high as 77%. Diabetes mellitus, peripheral vascular disease, neuropathy, and trauma are the most frequent causes of amputation [9]. The vitality of the soft tissues employed to obtain bone covering determines the extent of amputation [10,11].

Physiotherapy treatment for amputees is essential to enhancing their quality of life [12]. Phantom limb discomfort affects between 50% to 85% of amputees. It has been discovered that mirror therapy works well for treating phantom limb discomfort [13,14]. Following a lower limb amputation, physical therapy is started in the hospital while the patient is in acute care. Physiotherapists teach new amputees how to do various transfers and utilize a wheelchair and other mobility aids such as crutches and walkers [15]. All amputees want to be able to live a mobile, independent life. Most large lower limb ablations are performed for peripheral vascular disease, mostly affecting the elderly. As a result, additional medical diseases, social status, and accompanying extensive artery disease will likely exacerbate treatment issues. the amputee must adjust not only to using a prosthesis but also to a major shift in their perception of their body and, in certain cases, feelings akin to the death of a loved one. Hence, the patient already has enough difficulties without dealing with further technical complications from the amputation [16,17].

## Case presentation

Patient information

A 50-year-old male met with an RTA where he was hit by a vehicle from behind, resulting in injuries to his right leg with severe blood loss. The patient was conscious and was brought to the hospital on September 16, 2023, for which he was assessed; investigations included X-ray, which revealed comminuted fracture of the distal tibia and fibula. He then underwent open reduction external fixation surgery for the same on September 18, 2023. Subsequently, he noticed pus discharge over the operated part, indicating severe infection, and was managed with above-knee amputation on September 29, 2023. The patient was then referred for further physiotherapy management on October 5, 2023.

Clinical findings

Consent was obtained before assessment and treatment. The patient's built was ectomorphic. The patient was conscious, cooperative, and followed commands. On inspection, there was bandaging present over the right thigh. On palpation, skin covering the area appeared tense in the right leg. A diffuse swelling was noted over the proximal half of the left leg and knee. Grade 2 tenderness was present at the suture site (patient complains of pain and winces). The length of the bandage was 15 cm on examination, and superficial, deep, and cortical sensations above the level of amputation were intact. The hip range of motion (ROM) was accessed using a goniometer. Manual muscle testing (MMT) was conducted using the modified Medical Research Council scale.

Clinical investigations

**Figure 1:**
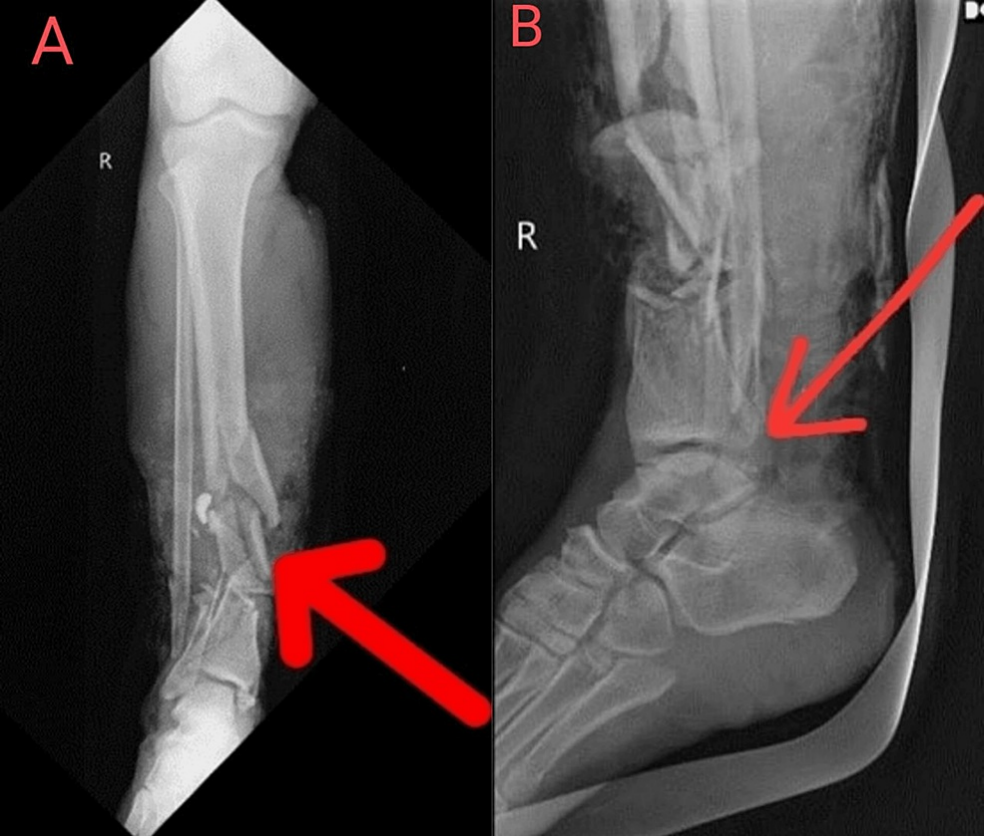
A. Right-sided comminuted displaced distal tibia and fibula fracture. B. Talus fracture at the foot (red arrow).

X-ray was performed, which revealed a right-sided displaced distal tibia and fibula fracture in the leg and a talus fracture at the foot (Figure 1).

**Figure 2:**
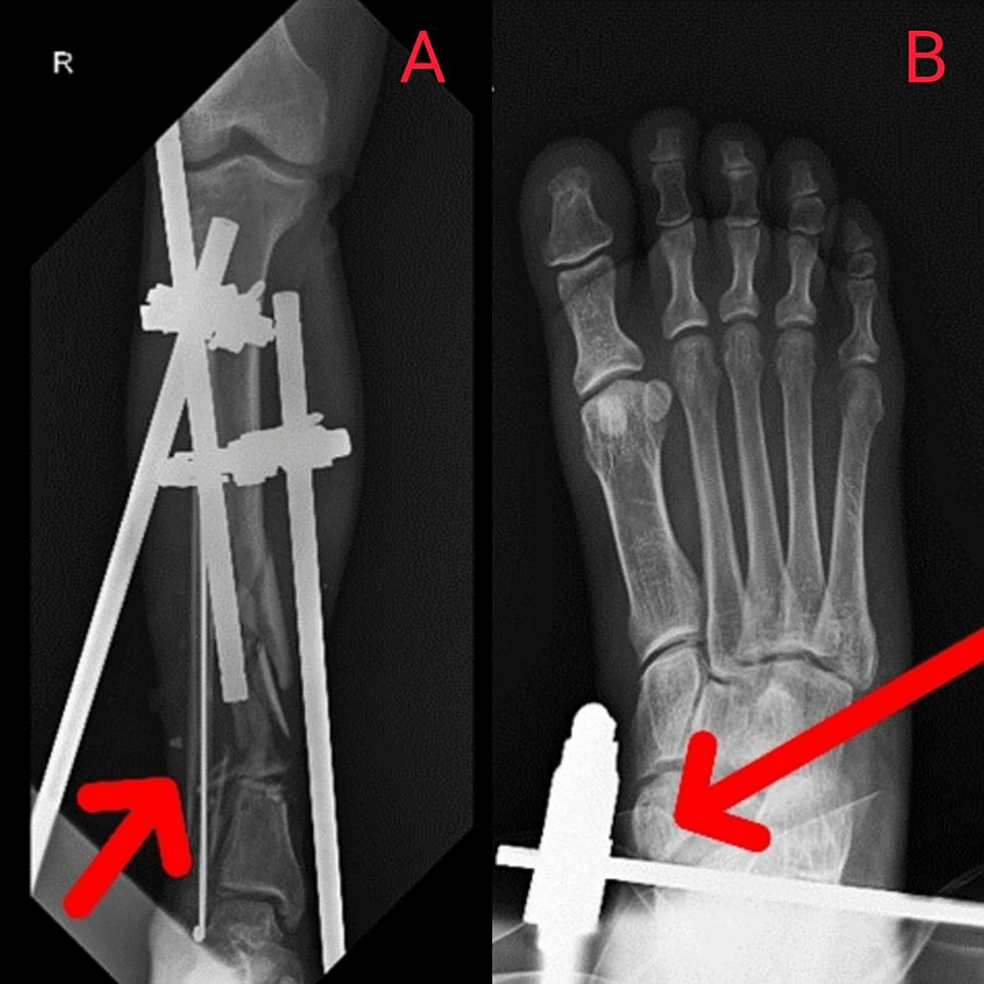
A: Right-sided comminuted displaced distal tibia and fibula fracture was managed with open reduction internal fixation. B. Talus fracture at the foot was managed with open reduction internal fixation (red arrow).

Figure 2 depicts X-ray after open reduction external fixation surgery.

**Figure 3:**
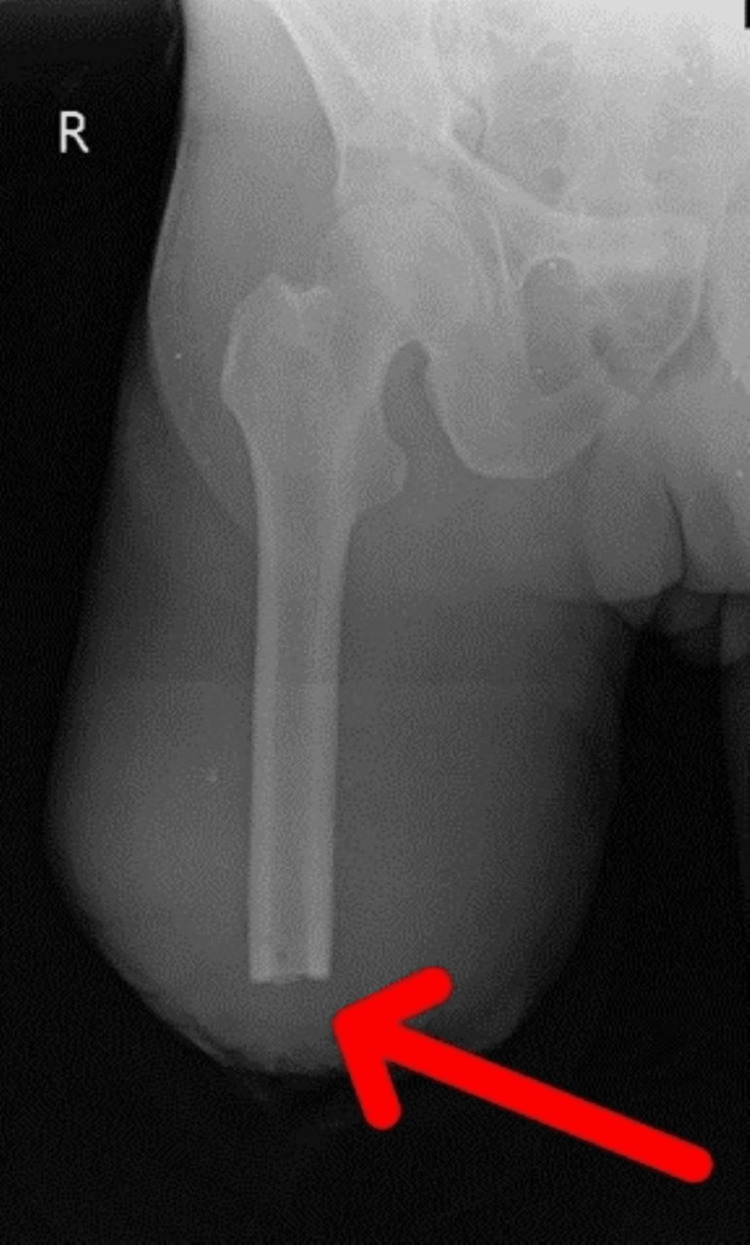
Post-operative X-ray showing above-knee amputation (red arrow).

Figure 3 shows the post-operative X-ray for above-knee amputation.

Physiotherapy management

During inpatient care, a four-week protocol was made with each session lasting for 60 minutes, twice daily, with appropriate rest intervals. The outcome measures were recorded before and after the completion of treatment. The treatment protocols are clearly defined in Table 1.

**Table 1:** Physiotherapy rehabilitation protocol ADL, activities of daily living; IRR, infrared radiation; reps, repetition; ROM, range of motion

Goals	Interventions	Exercise dose
Psychosocial support and education	Counseling and emotional support were offered in various settings, accompanied by peer support. Additionally, education on the utilization and maintenance of prosthetics was provided.	Regular sessions based on patient needs
Reducing pain and controlling edema	RICE Protocol was used to reduce pain and swelling. Rest: rest was recommended for the amputated residual limb throughout the duration, with the exception of exercise periods. Ice: application of ice in form of ice pack to the affected area was advised for duration of 5-7 minutes to reduce pain. Compression: compression bandage was applied to the involved extremity to reduce edema. Elevation: elevated position of residual limb was advised to control edema.	2-3 times a day
Promoting healing and improvinf scar mobility	IRR; scar massage and desensitization	As per wound condition, 2-3 times a week
To prevent chest complications	Deep breathing exercises; thoracic expansion exercises	10 reps x 1 set
Preventing contracture	Encourage the stretching exercises for hip adductors, hip abductors, hip flexors, and hip extensors. Additionally, advocate for 20 minutes of prone lying to prevent the development of flexion contracture.	10 reps x 1 set
Restoring ROM	Passive and active ROM exercises for bilateral hip joint and left knee joint	2-3 times a day, 10 reps x 1 set
Increasing strength of muscles	Isometric exercises targeting the hip flexors, extensors, adductors, and abductors were performed during first two weeks. Weeks 3 and 4 were focused on strengthening the residual limb using a resistance band for the hip flexors, extensors, adductors, and abductors.	3-4 times a week, 10 reps x 1 set
Strengthening of upper limb muscles	Strengthening of upper limb muscles using 1 kg of weight cuff	10 reps x 1 set
Mobility and transfers	Bed mobility exercises; transfer training (bed to chair, chair to toilet); ambulation with an assistive device (walker)	Daily progressively increasing duration
Improving balance and coordination	Balance exercises on stable and unstable surfaces; proprioceptive training; gait training with an assistive device (walker)	3-4 times a week, 20-30 minutes/session
Independence in ADLs	Training in self-care activities (dressing, bathing, grooming); adaptive equipment assessment and training; home modification recommendations	Integrated into daily therapy sessions

**Figure 4:**
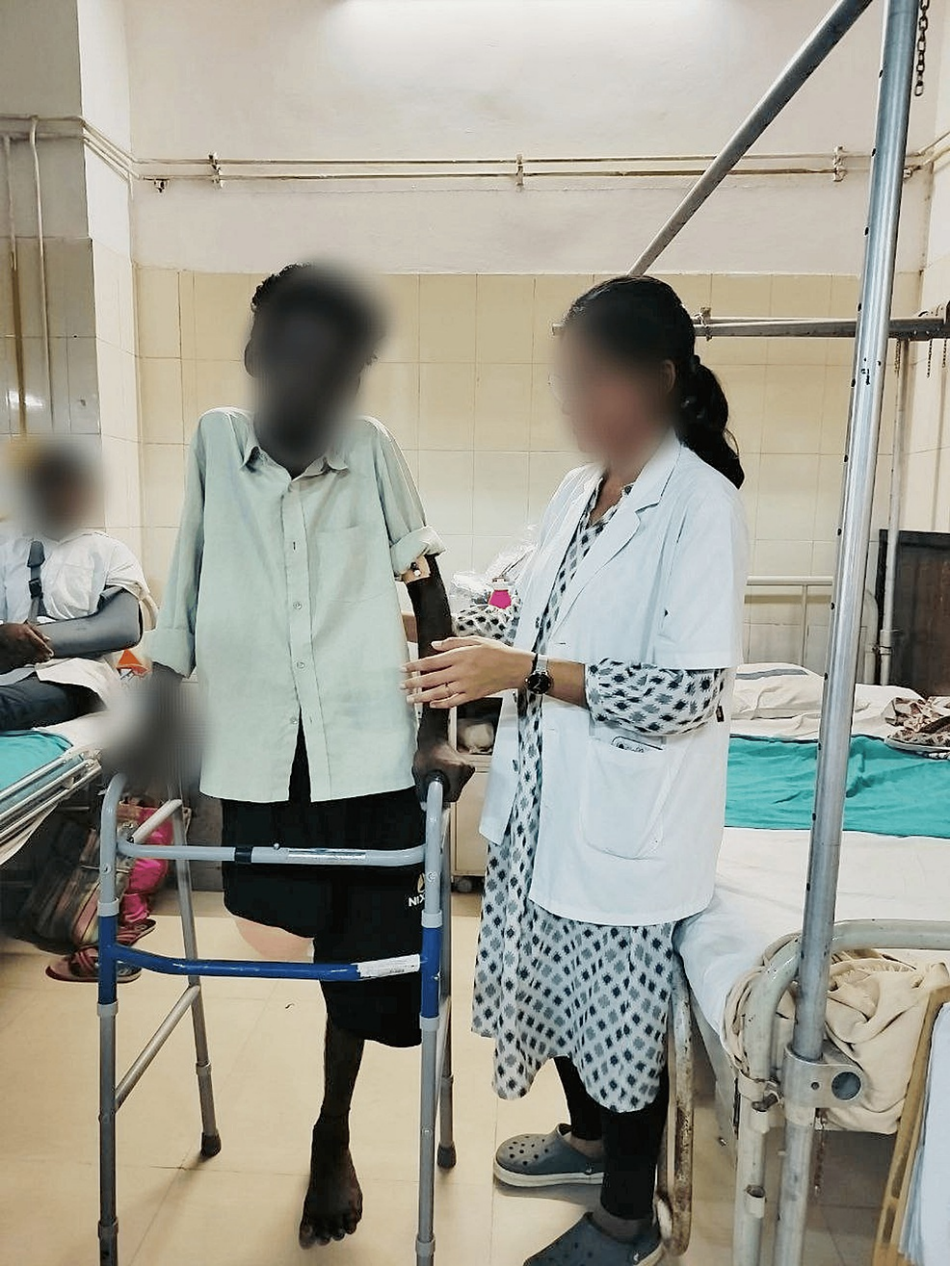
Post-operative gait training with a walker.

**Figure 5:**
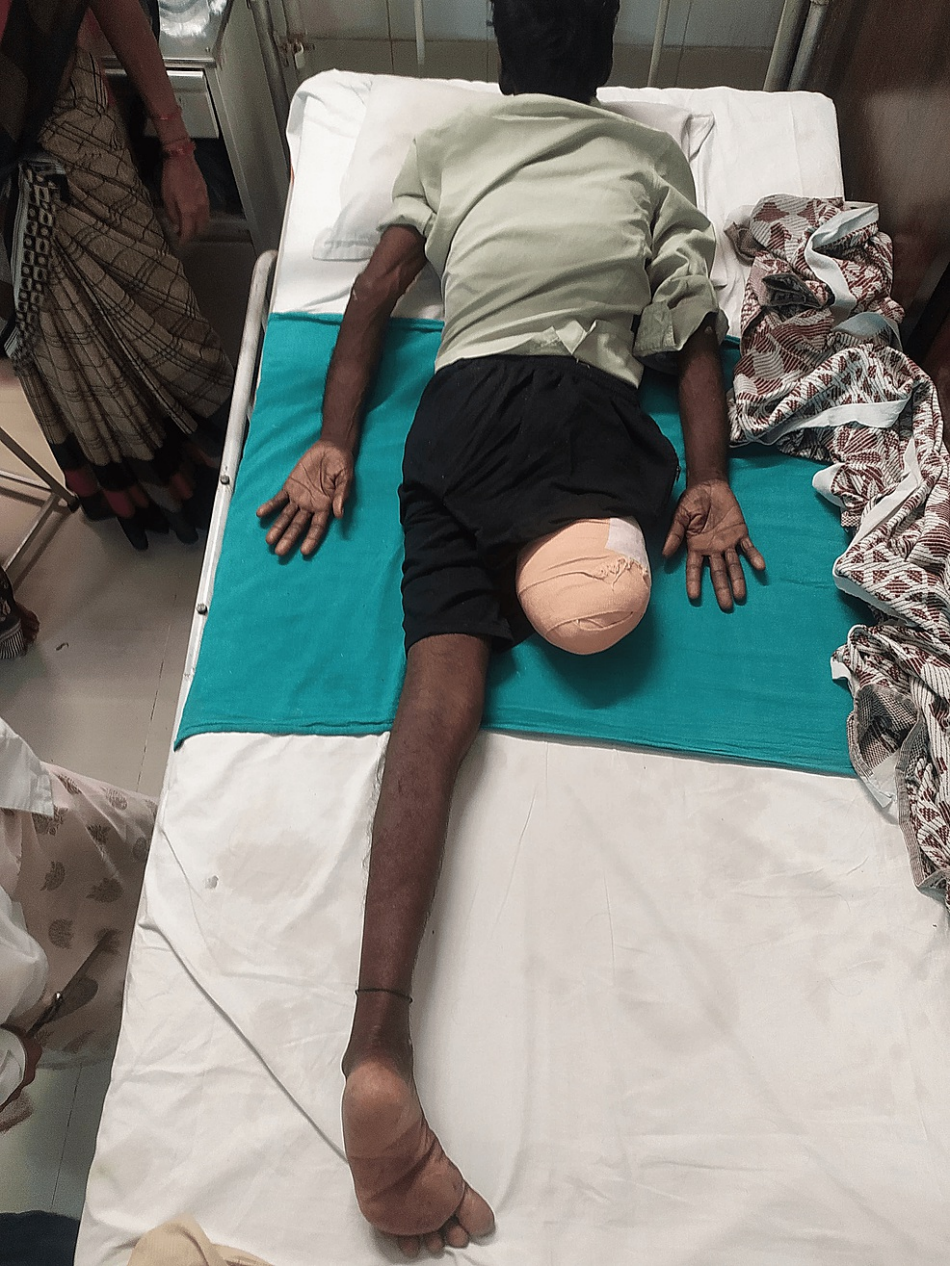
Post-operative image showing the patient in the prone lying position.

**Figure 6:**
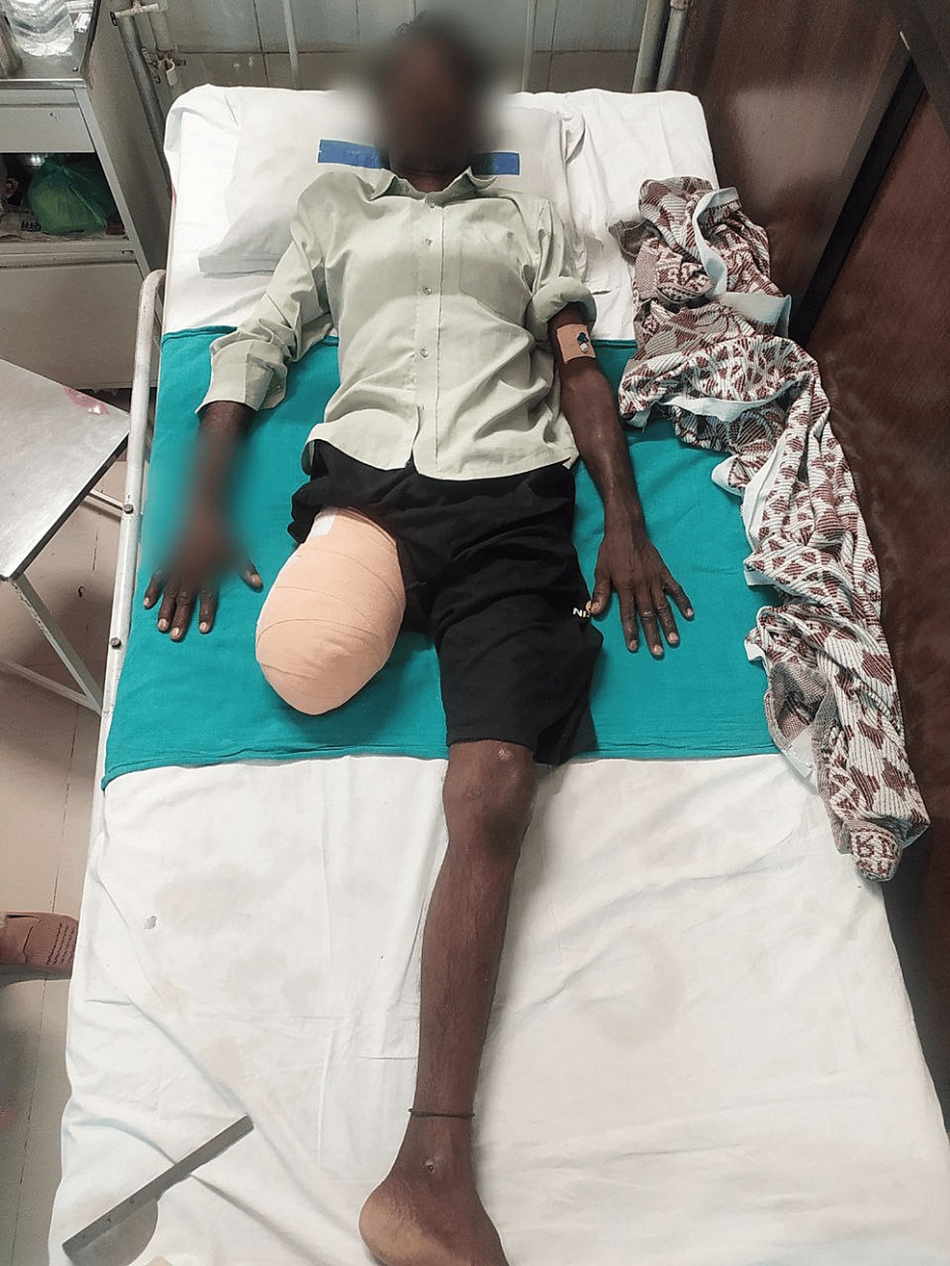
Post-operative image showing the patient in the supine position.

**Figure 7:**
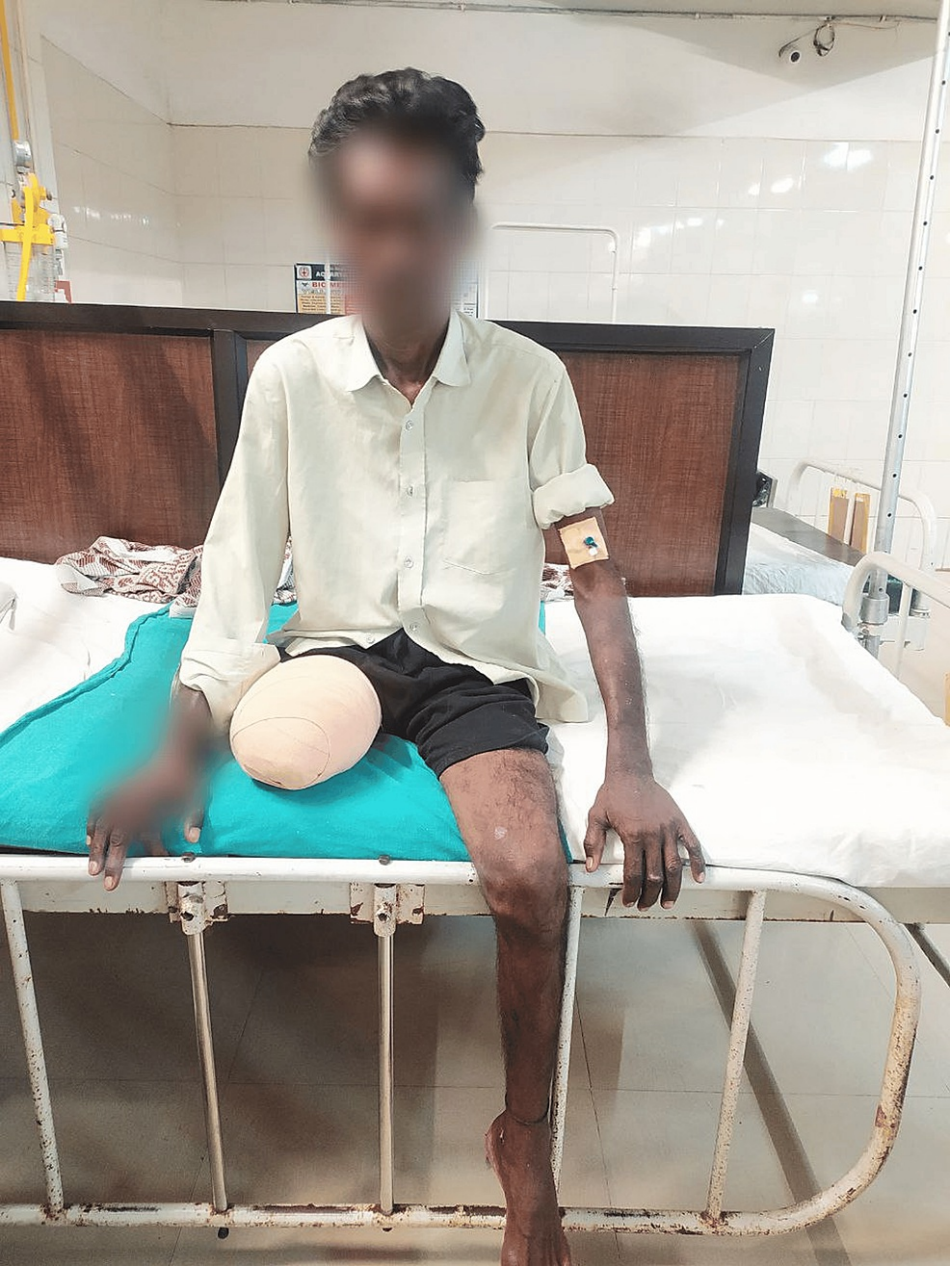
Post-operative image showing the patient in the sitting position.

Figure 4 shows the patient performing ambulation with the assistance of a walker. Figure 5 shows the patient in the prone lying position. Figure 6 shows the patient in the supine lying position. Figure 7 shows the patient in the sitting position.

Outcome measures 

Table 2 shows the assessment findings for MMT. Table 3 shows the assessment for outcome measures: functional independence measure and visual analog scale. Table 4 summarizes the ROM examination findings.

**Table 2:** Improvement in MMT before and after the treatment Note: 3 indicates full range of motion against gravity, 4 indicates full range of motion against gravity with minimal resistance, 5 indicates full range of motion against gravity with maximal resistance, 2 indicates full range of motion in gravity eliminated plane MMT, manual muscle testing

MMT	Pre-treatment		Post-treatment	
Hip joint	Right	Left	Right	Left
Flexors	2/5	3/5	3/5	4/5
Extensors	2/5	3/5	3/5	4/5
Adductors	2/5	3/5	3/5	4/5
Abductors	2/5	3/5	3/5	4/5

**Table 3:** Outcome measures

Outcome measures	Pre-treatment	Post-treatment
Functional independence measure	33/126	80/126
Visual analog scale	8/10	3/10

**Table 4:** Improvement in ROM before and after the treatment ROM, range of motion

Range of motion	Pre-treatment				Post-treatment			
Hip joint	Right (active)	Right (passive)	Left (active)	Left (passive)	Right (active)	Right (passive)	Left (active)	Left (passive)
Flexion	20^°^	28^°^	60^°^	90^°^	50^°^	65^°^	80^°^	110^°^
Extension	10^°^	12^°^	20^°^	24^°^	20^°^	23^°^	30^°^	30^°^
Adduction	10^°^	14^°^	20^°^	30^°^	25^°^	30^°^	30^°^	30^°^
Abduction	20^°^	26^°^	30^°^	40^°^	40^°^	45^°^	45^°^	45^°^

## Discussion

In this case, a 50-year-old man met with RTA, suffering from severe injuries to his right leg and severe bleeding. He was then taken to hospital for treatment for the same. Investigations included X-ray, which revealed that he had right-sided displaced distal tibia and comminuted fibula fracture and talus fracture at the foot. He underwent open reduction external fixation surgery for the same. He thereafter observed pus flow over the surgical site, indicating a serious infection, for which he underwent above-knee amputation. The patient was then referred by the orthopedic surgeon to a physiotherapist for further rehabilitation. We planned a physiotherapy protocol in which we concentrated on enhancing muscular strength, reducing phantom limb pain, avoiding secondary complications, and preserving ROM, endurance, and functional independence in the postoperative phase. After an amputation, balance and posture training are crucial in assisting the amputee to regain their independence. Numerous investigations have been conducted to discover new developments in these situations. A research was conducted in 2012 to examine the impact of video games on balance training during rehabilitation for children and adolescents with lower limb amputations. The results were promising, but the long-term implications were only partially evident [18].

In our instance, we trained the patient's gait and balance using traditional physiotherapy techniques, which enhanced the total functional independence. An investigation conducted in 2007 found that brief, intense physical therapy rehabilitation improves walking speed and other functional abilities [19]. Since improving the patient's total independence was our primary goal, the patient underwent balancing and gait training, and information about prosthesis was also given. An analysis of the literature published in 2018 concluded that applying physiotherapy positively impacted functional status. A proper prosthesis and early physiotherapy rehabilitation considerably enhance functional results in research. Lower energy consumption, improved balance, and normalization of gait patterns were seen [20]. In this case, there was a remarkable improvement in the patient’s functional activity. He became more confident as well.

## Conclusions

According to the data, the patient's functional independence and physical health showed notable improvements. After therapy, the patient showed improvements in total functional independence, muscular strength, and ROM. The research emphasizes the beneficial effects of physiotherapy interventions that were put into place on the patient's mobility, self-sufficiency, and quality of life after the amputation and subsequent rehabilitation. The interventions focused on pain management, wound healing, mobility training, and psychosocial support, leading to remarkable improvements in the patient's ROM, muscle strength, and overall functional independence. The successful outcomes documented in this study provide valuable insights into the effective utilization of physiotherapy, facilitating the patient's transition to a more self-sufficient and active lifestyle after limb loss. This research contributes to the existing body of knowledge, highlighting the essential role of physiotherapy in post-amputation treatment plans and emphasizing the potential for enhanced quality of life, mobility, and self-sufficiency among amputees. These results facilitate the patient's transition to a more independent and active lifestyle by offering effective integration of physiotherapy in the multidisciplinary approach to post-amputation treatment.
